# Correction: Physical probing of quantum energy levels in a single indium arsenide (InAs) quantum dot

**DOI:** 10.1039/d4na90068e

**Published:** 2024-06-17

**Authors:** Moh'd Rezeq, Yawar Abbas, Boyu Wen, Zbig Wasilewski, Dayan Ban

**Affiliations:** a Department of Physics, Khalifa University of Science and Technology POB 127788 Abu Dhabi United Arab Emirates mohd.rezeq@ku.ac.ae; b System on Chip Centre, Khalifa University of Science and Technology POB 127788 Abu Dhabi United Arab Emirates; c Department of Electrical and Computer Engineering, Waterloo Institute for Nanotechnology, University of Waterloo ON Canada dban@uwaterloo.ca

## Abstract

Correction for ‘Physical probing of quantum energy levels in a single indium arsenide (InAs) quantum dot’ by Moh'd Rezeq *et al.*, *Nanoscale Adv.*, 2023, **5**, 5562–5569, https://doi.org/10.1039/D3NA00638G.

The authors regret that in the original publication, eqn 2(a)–(c) contained errors. The correct equations are given below:2a
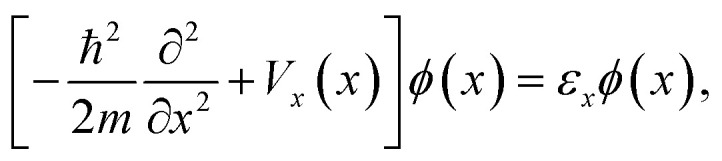
2b
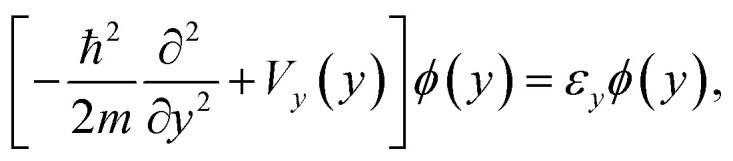
2c
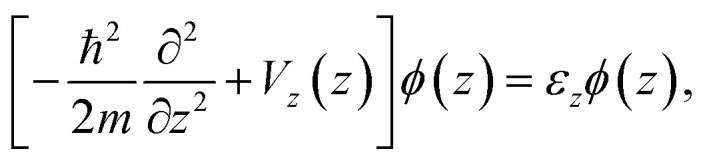


In addition, in the first line of the Abstract, the abbreviation for molecular beam epitaxy is incorrectly listed as EBM instead of the correct abbreviation MBE. Lastly, in the first line on page 5565 in the original publication, there is a typographical error in the word “indicate…”. The correct sentence is the following: “This indicates a completely discharged QD.”

The Royal Society of Chemistry apologises for these errors and any consequent inconvenience to authors and readers.

